# A Case of Fragility of the Bones

**Published:** 1785

**Authors:** 


VII. *A Caſe of Fragility of the Bones. By Mr.* W. Goodwin, *Surgeon at Earl-Soham, in Suffolk. Communicated in a Letter to Dr.* Hamilton, *Phyſician at Ipſwich; and by him, with ſome additional Obſervations, to Dr.* Simmons.

MARY Bradcock, a poor woman of the pariſh of Dalinghoe, near Wickham market, in Suffolk, in the ſevere winter of 1783 was ſeized with pain in moſt of her limbs, which ſhe attributed to rheumatiſm, when one day, walking acroſs the houſe, ſhe tripped her foot againſt a brick, and was much ſurpriſed to find her leg broken near the ancle.

Before

Before ſhe was perfectly recovered from this accident ſhe became pregnant, and, growing infirm and weak, was one day aſſiſted by her huſband in getting out of bed, when her left thigh bone ſnapped aſunder, though no particular force had been exerted.

She was ſafely delivered, and, ſoon after, her left arm was fractured near the ſhoulder, merely by putting it over the neck of an aſſiſtant to raiſe herſelf in bed. This likewiſe formed a callus, and grew well. She next found her right thigh bone broken as ſhe lay in bed, very high up towards the hip, as it was, likewiſe, ſome time after, lower down near the knee. One of her collar bones has alſo ſeparated without violence. Her right arm has met with a like misfortune by lifting a pint baſon from a table.

She now lays with the third fracture of her right thigh, which happened laſt Sunday, (from her being gently raiſed in her bed) at or near the part by the knee which was before broken and united by callus.

The bones are permitted to grow together in an irregular manner, with the aſſiſtance only of bathing and bandage, as an extenſion of the limbs would be dangerous; for ſo deplorable is her ſituation, that ſhe cannot venture to be moved even to have her bed made, for fear of breaking her bones.

She is thirty-two years old, of a delicate make, lax fibre, fair complexion, and pale brown hair. She is at preſent in the fixth month of her ninth pregnancy; has always been temperate in her manner of living; has never taken mercurial medicines; and has, in general, enjoyed a good ſtate of health.

Before the bones break ſhe conſtantly feels a conſiderable pain, for ſeveral weeks, on the very ſpot where the fracture is to take place. This pain continues to increaſe till the bone breaks, and then goes off in a few days, and the bone unites in five or ſix weeks by forming a callus. She now complains of pain a little above one of her elbows, and, from what ſhe has ſo repeatedly experienced, expects that her arm will break at that part.

This unhappy woman has had eight fractures within the ſpace of a year and a half, ſeven of which have happened in the courſe of the laſt twelve months, and all without any ſufficient, or, indeed, any external cauſe to which they could be attributed.

*Earl-Soham,*

*near Framlingham, in Suffolk,*

Auguſt 7, 1785.

*Additional*

### *Additional Obſervations on the preceding Caſe, by Dr.* Hamilton.

I have made the inquiries you recommended to me relative to the caſe I lately tranſmitted to you. I have alſo viſited the patient, as hundreds of late have done, in conſequence of her caſe having been deſcribed in the Ipſwich Journal to excite the charitable to ſend her donations. In the ſtate of her urine and perſpiration I could diſcover nothing different from health. From her complexion and other circumſtances one would be inclined to ſuſpect a diſpoſition to ſcrophula, though ſhe has never had the diſeaſe. She informs me, however, that ſeveral of her family have been afflicted with it, and one of her children now labours under it.

Her right thigh is conſiderably diſtorted, which is owing to the irregular manner in which it has been neceſſary to ſuffer the fractured ends of the bone to unite. Her left thigh is at leaſt twice as large as the right, which is perhaps occaſioned by preſſure on the lymphatics of that limb, as ſhe lies conſtantly on her left ſide.

To the above, and other inſtances of a brittleneſs of the bones, which are to be met with in books, give me leave to add that of a lady of this town, who, after having been for a conſiderable time afflicted with a cancer of the breaſt, of which ſhe died, had her thigh fractured one day merely by riſing from her ſeat.

*Ipſwich,*
Auguſt 28, 1785.

